# Supplementary material for: Divergent sequences of tetraspanins enable plants to specifically recognize microbe-derived extracellular vesicles
Source: Nat Commun. 2023 Aug 12;14:4877. doi: 10.1038/s41467-023-40623-0 (PMC10423219; doi:10.1038/s41467-023-40623-0)
Supplement: Supplementary file 1 — Supplementary Information [file 41467_2023_40623_MOESM1_ESM.pdf]

Supplementary Information

Divergent sequences of tetraspanins enable plants to specifically recognize microbe-derived extracellular vesicles

Jinyi Zhu[1], Qian Qiao[1], Yujing Sun[1], Yuanpeng Xu[1], Haidong Shu[1], Zhichao Zhang[1], Fan Liu[1], Haonan Wang[1], Wenwu Ye[1,2,3], Suomeng Dong[1,2,3], Yan Wang[1,2,3], Zhenchuan Ma[1,2,3], Yuanchao Wang[1,2,3,] *

[1]Department of Plant Pathology, Nanjing Agricultural University, Nanjing 210095, China

[2]The Key Laboratory of Integrated Management of Crop Diseases and Pests (Ministry of Education), Nanjing 210095, China

[3]The Key Laboratory of Plant Immunity, Nanjing Agricultural University, Nanjing 210095, China

*Yuanchao Wang (corresponding author)

Email: wangyc@njau.edu.cn

Supplementary Figures

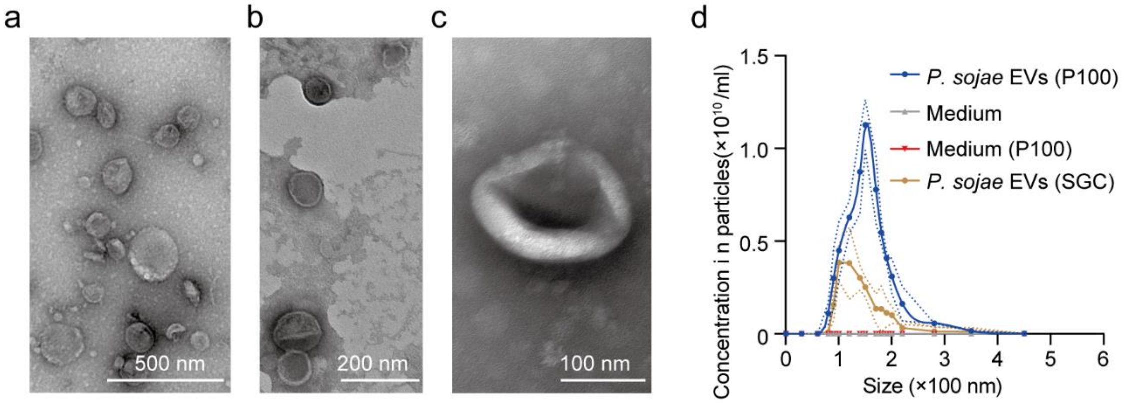

Supplementary Fig. 1 Morphology and size of EVs released by *P. sojae*. a, b, c Negative staining and transmission electron microscopy of EVs from *P. sojae* culture fluid show vesicle-like structures. **a** Morphology of EVs in the P100 fraction (pellet of 100,000 g centrifugation). **b** Morphology of EVs purified using sucrose gradient centrifugation. **c** Morphology of a single EV. **d** Nanoparticle tracking analysis results showing the size of EVs isolated from *P. sojae* culture fluid of the P100 fraction and the EVs after sucrose gradient centrifugation (SGC) and the P100 fraction of medium and medium itself. Mean values (±SD shown as dotted lines) of three replicates are shown**.** The experiments were repeated three times with similar results. Source data are provided as a Source Data file.

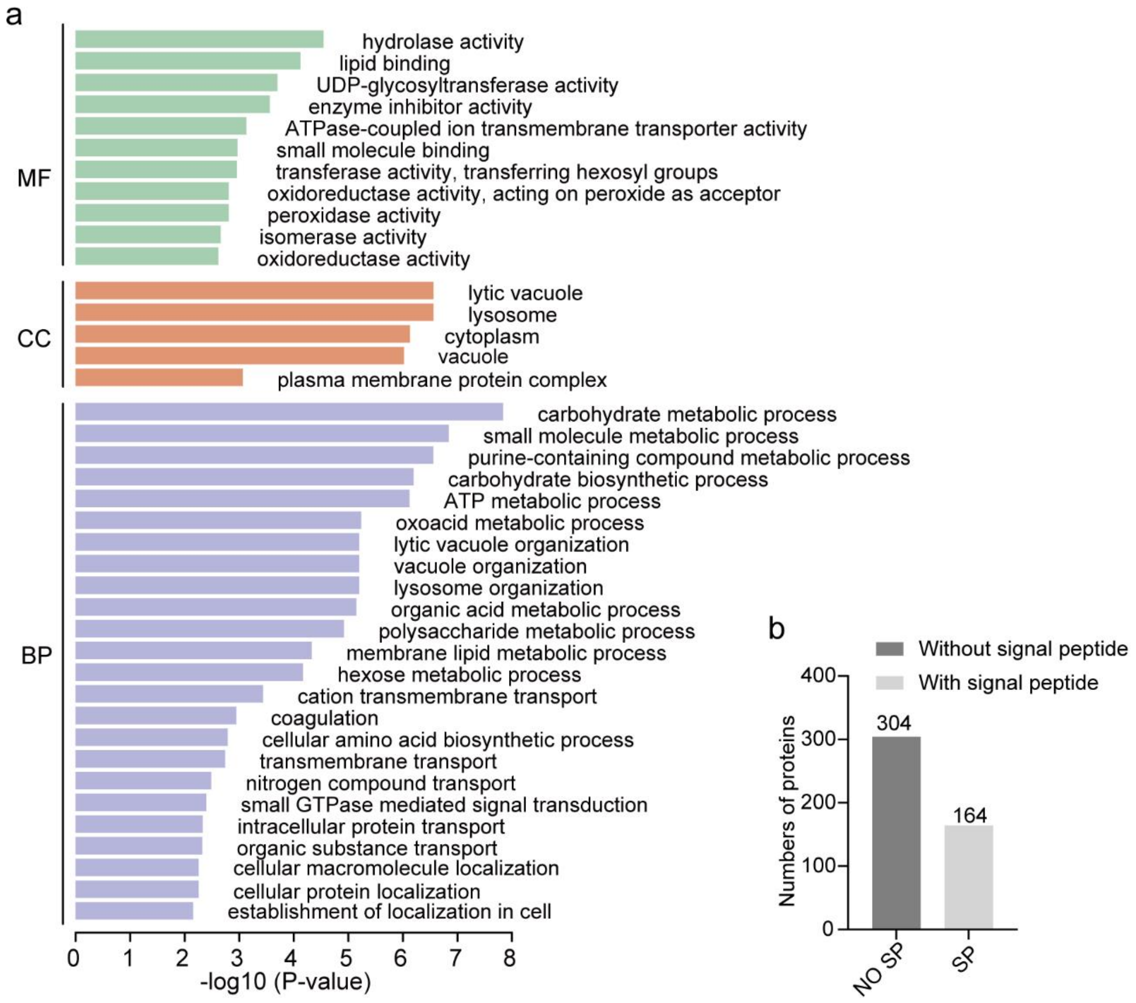

Supplementary Fig. 2 Gene ontology (GO) enrichment analysis of proteins enriched in EVs compare to whole proteomic. a GO enrichment analysis of *P. sojae* EV proteins compared to overall proteome. The x coordinate represents the adjusted p-value, the y coordinate represents the GO terms of molecular function (MF), cellular component (CC), and biological process (BP) of enriched genes. **b** Signal peptide prediction of EV proteins. The signalP 4.1 server determined prediction results. SP indicates the sequences predicted to have a signal peptide. NO SP indicates that the sequences were predicted not to have a signal peptide. Source data are provided as a Source Data file.

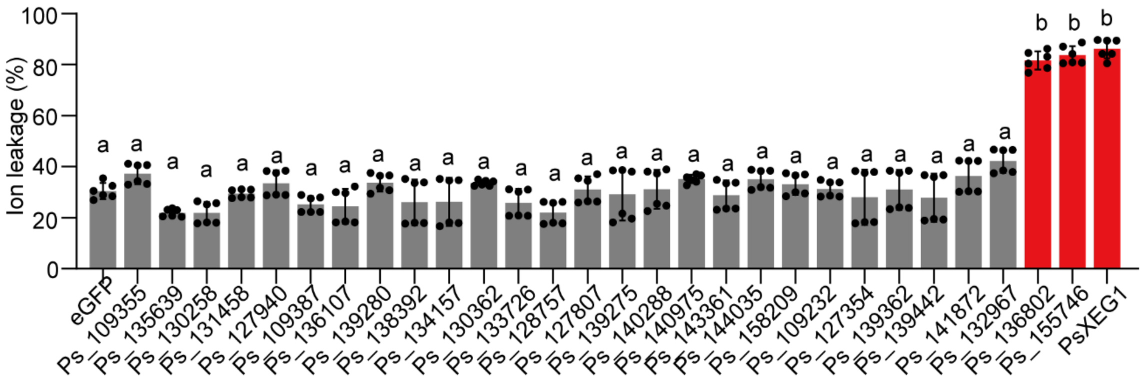

Supplementary Fig. 3 Ion leakage assays. Quantification of cell death using ion leakage assays of leaf discs taken after 3 dpi. Mean values (±SD) of six measurements are shown. Different letters represent significant differences (P< 0.0001; one-way ANOVA). For exact *p* values, see source data. The experiments were repeated three times with similar results. Source data are provided as a Source Data file.

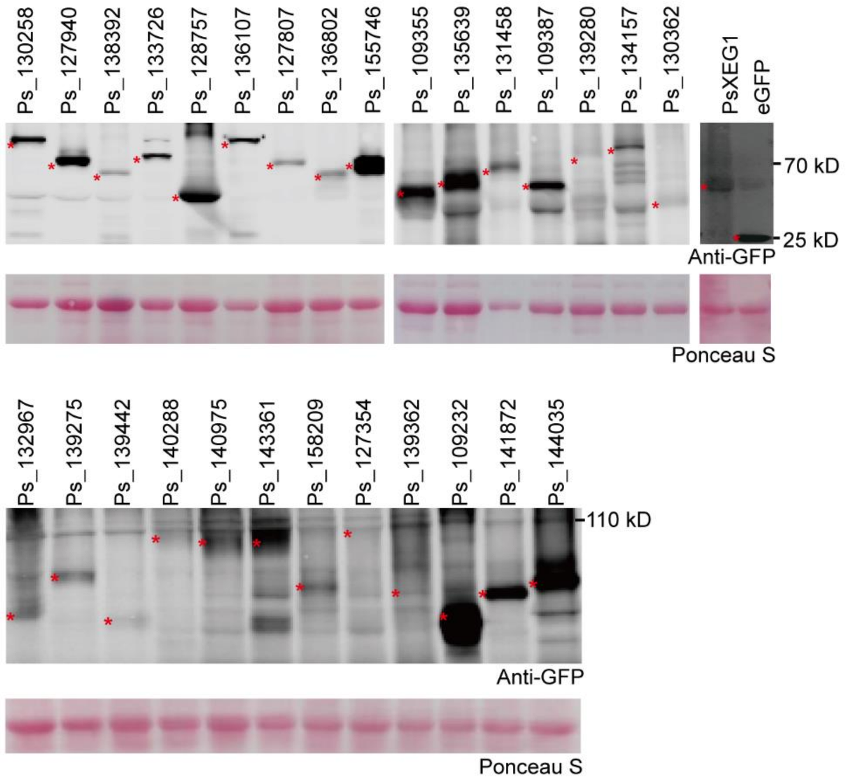

Supplementary Fig. 4 Accumulation of candidate EV transmembrane proteins transiently expressed in *N. benthamiana*. Immunoblot analysis of transiently expressed proteins fused with an eGFP tag at the N terminus. Total proteins were extracted from *N. benthamiana* leaves 2 days after agro-infiltration. Ponceau S-stained Rubisco protein is shown as a total protein loading control. Red stars indicate expected sizes. The experiments were repeated three times with similar results. Source data are provided as a Source Data file.

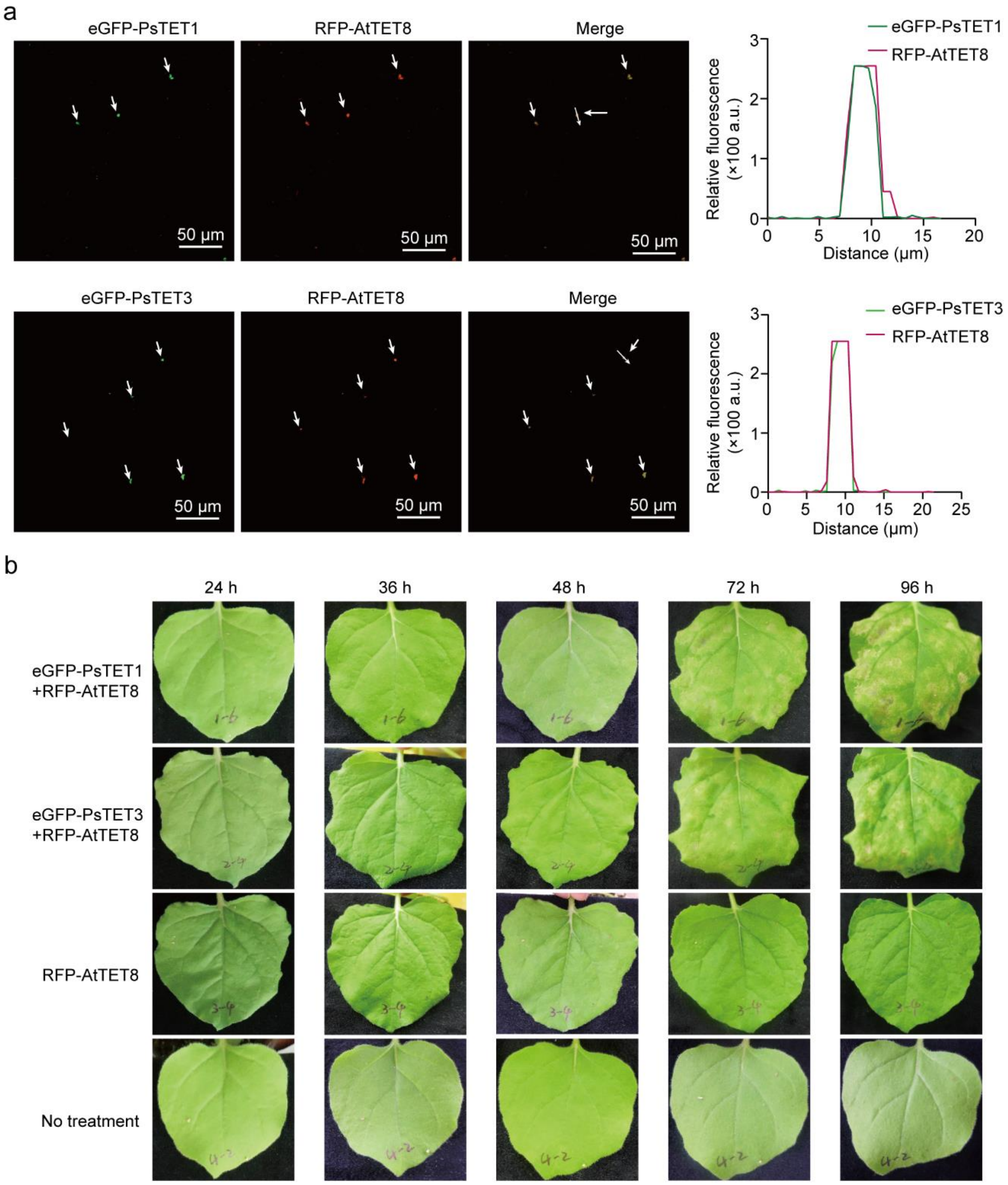

Supplementary Fig. 5 *P. sojae* PsTET1 and PsTET3 proteins colocalized with EV maker AtTET8 in isolated EVs. a eGFP-PsTET1 or eGFP-PsTET3 was co-expressed transiently with RFP-AtTET8 in *N. benthamiana*. Confocal microscopy was used to determine the localization of PsTETs with AtTET8. **b** Representative leaves showing plant

condition after expression of the indicated proteins in *N. benthamiana* leaves. EVs were isolated before 48 hours after agro-infiltration, prior to the development of cell death. Leaves (n=6) were photographed at different time point after agro-infiltration. All experiments were repeated three times with similar results. Source data are provided as a Source Data file.

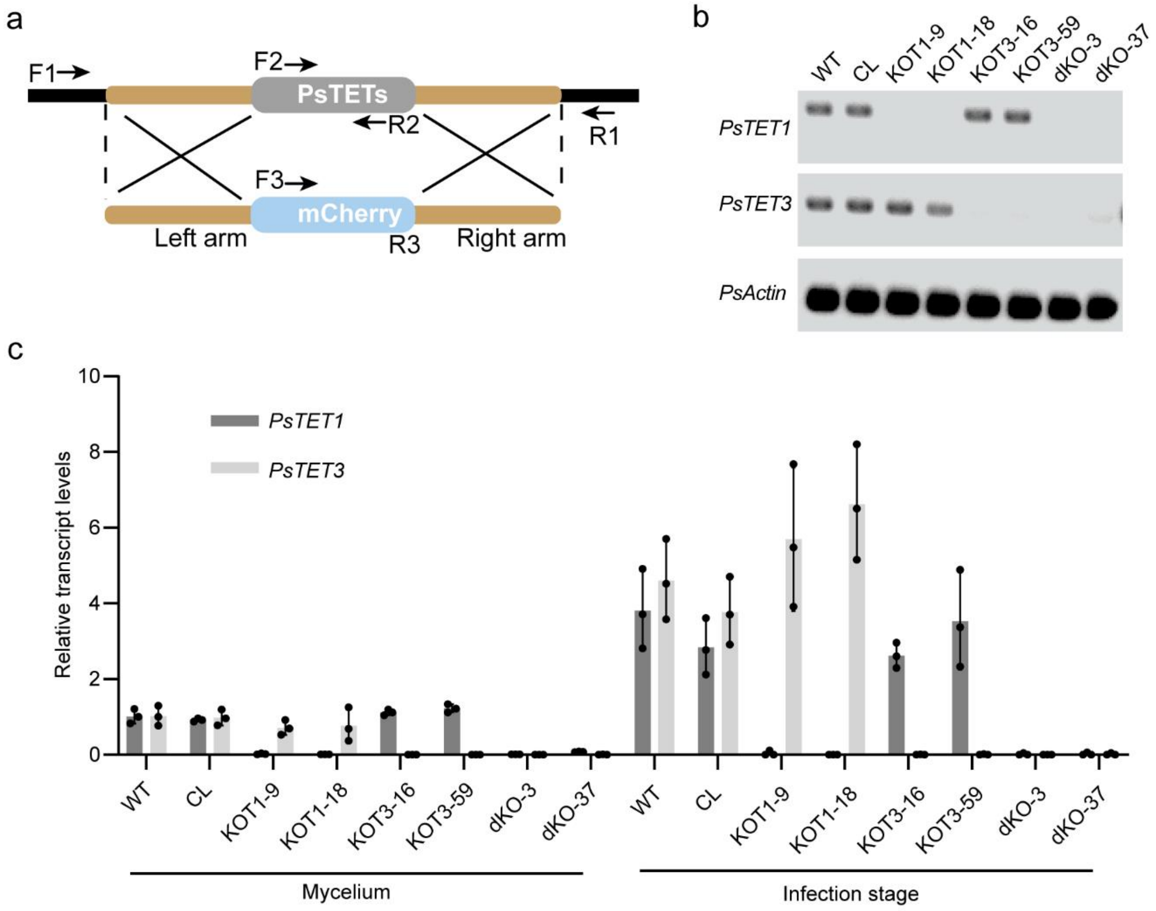

Supplementary Fig. 6 Validation of *P. sojae PsTET1* and *PsTET3* knockout mutants. **a** Schematic representation of the targeted gene knockout and replacement by *mCherry* in *P. sojae* using CRISPR/Cas9. The indicated primers were used for screening the positive transformants and are listed in Supplementary Table 4. **b** RT-PCR assay to verify the *P. sojae* knock out transformants. The RNA was extracted from the transformants and RT-PCR was performed using the primers listed in Supplementary Table 4. **c** Relative transcript levels of *PsTET1* and *PsTET3* in the knock out transformants. Total RNA was extracted from transformants of mycelium and infection stage. Relative transcript levels were determined by RT-qPCR. *PsActin* was used as the internal reference gene. Levels of *PsTET1* and *PsTET3* were normalized to *PsActin* then set relative to the levels of the wild type (set to 1). Mean values (±SD) of three replicates are shown. All experiments were repeated three times with similar results. Source data are provided as a Source Data file.

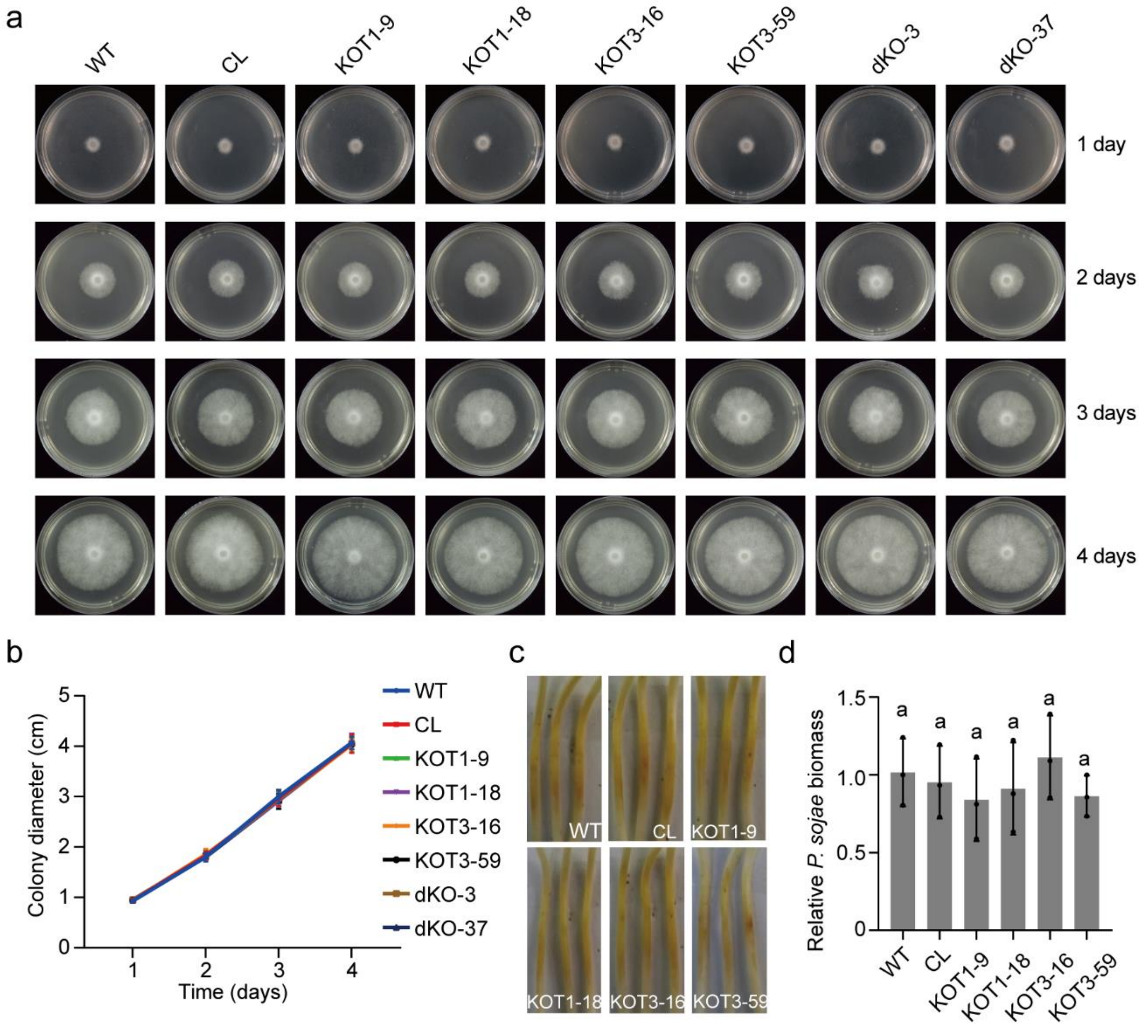

Supplementary Fig. 7 Characterization of P. sojae *PsTET1* and *PsTET3* knockout mutants. a Growth characteristic of the wild-type (P6497), *PsTET1* knockout mutants (KOT1-9, KOT1-18), *PsTET3* knockout mutants (KOT3-16, KOT3-59), *PsTET1* and *PsTET3* double knock out mutants (dKO-3, dKO-37) and failed knockout control line (CL) during 4 days growth on V8 medium. **b** Colony diameter of the indicated strains grow on V8 medium, measured from 1 day to 4 days after transfer. Mean values (±SD) of six measurements are shown. No significant differences were found by one-way ANOVA. For exact *p* values, see source data. **c** Virulence of *P. sojae PsTET1* and *PsTET3* single knockout mutants does not differ from wild type. Etiolated soybean hypocotyls were inoculated with zoospore suspensions from the indicated strains. Disease symptoms were photographed at 2 days post-inoculation. **d** Relative pathogen biomass in inoculated

etiolated hypocotyls measured as the ratio between the amounts of *P. sojae* DNA and soybean DNA assayed at 2 dpi by qPCR; the P6497/soybean ratio was set at 1.0. Mean values (±SD) of three replicates are shown. No significant differences were found by one-way ANOVA. For exact *p* values, see source data. All experiments were repeated three times with similar results. Source data are provided as a Source Data file.

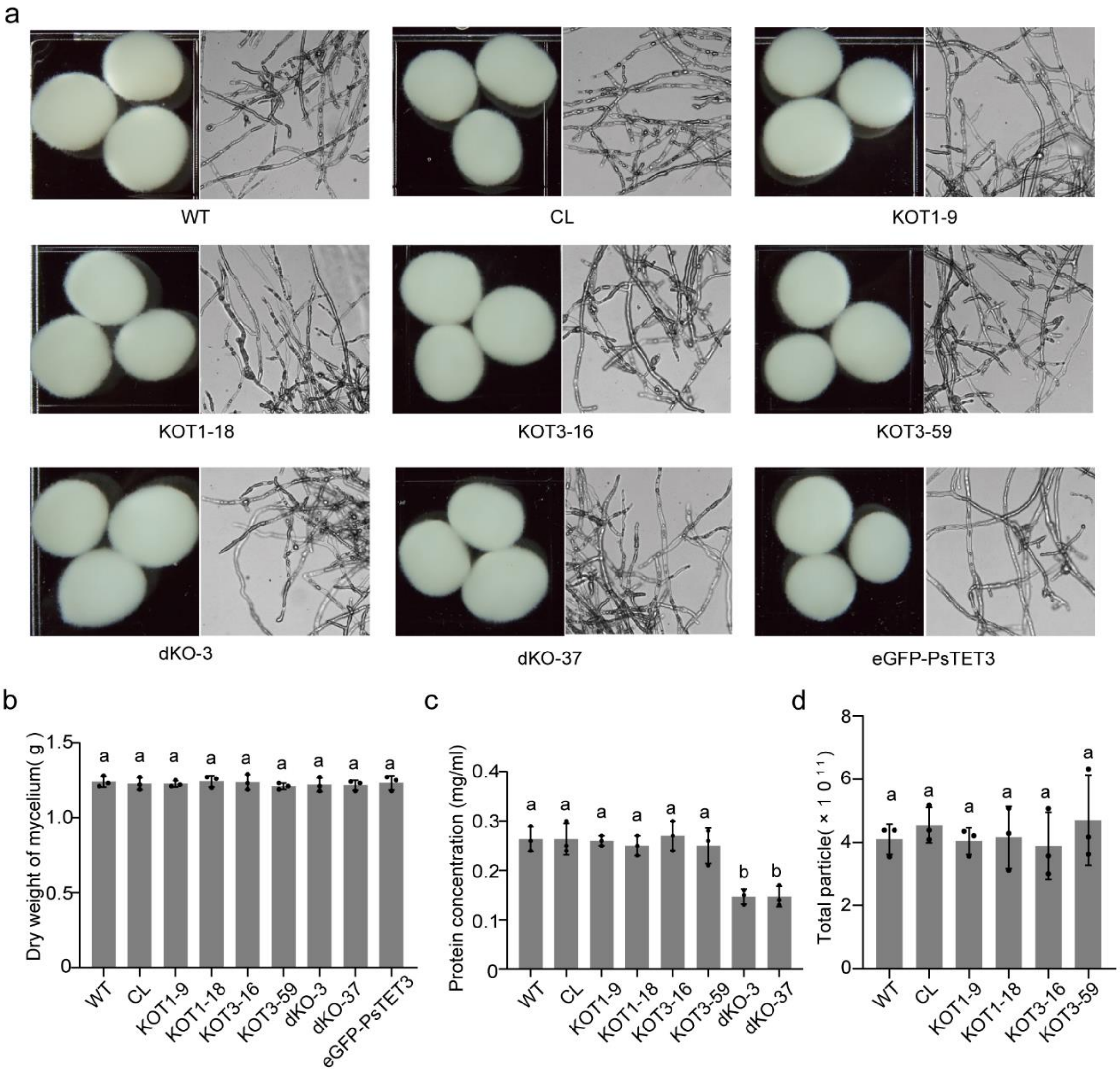

Supplementary Fig. 8 The growth behavior of *P. sojae* transformants in liquid medium. a Morphology of mycelium balls and mycelium. *P. sojae* wild type and transformants are cultured in liquid medium. mycelium balls and mycelium were photographed after 8 days cultivation. **b** Mycelium balls were collected after 8 days cultivation and measured the dry weight. Mean values (±SD) of three replicates are shown. No significant differences were found by one-way ANOVA. **c** Protein concentration of EVs by knockout mutants and wild type. Mean values (±SD) of three replicates are shown. Different letters represent significant differences ($P< 0.001$, one-way ANOVA). **d** NTA analysis of EV levels released into culture media by knockout mutants and wild type. Mean

values (±SD) of three replicates are shown. No significant differences were found by one-way ANOVA. For exact p values, see source data. All experiments were repeated three times with similar results. Source data are provided as a Source Data file.

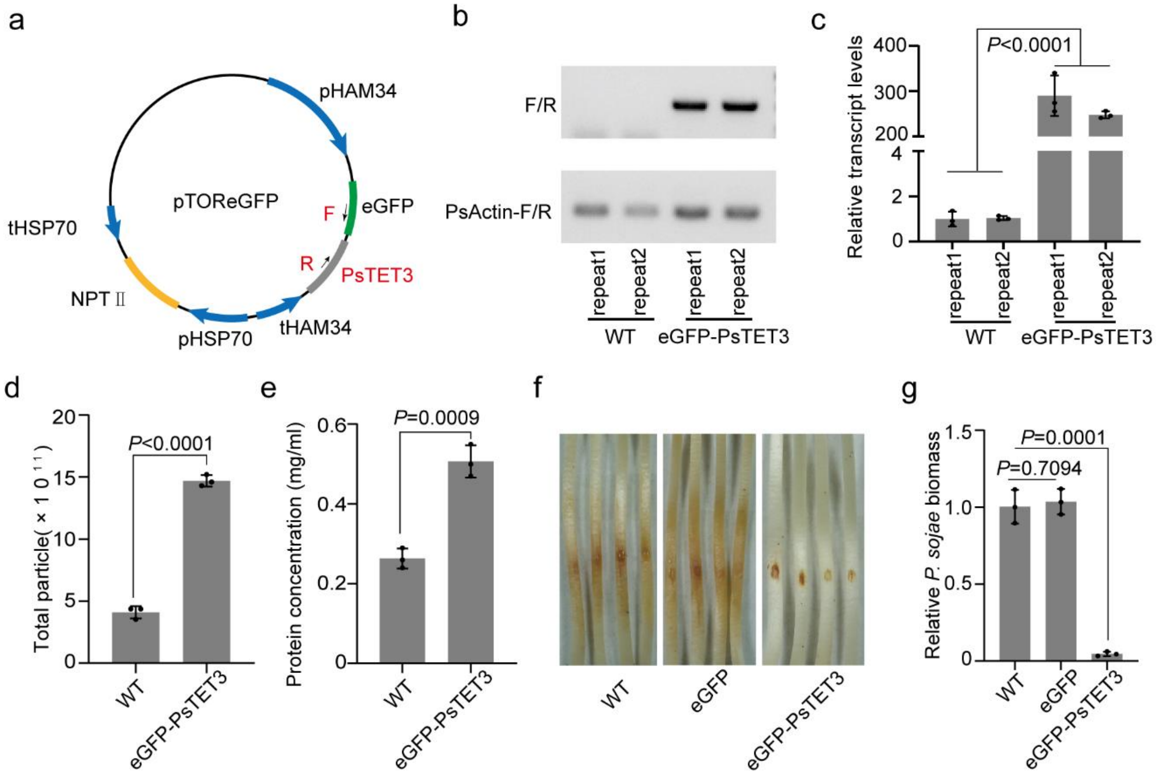

Supplementary Fig. 9 Isolation of the eGFP-PsTET3 overexpression line of *P. sojae*. a Schematic diagram showing the plasmid used for the *P. sojae* transformation. **b** PCR assay to verify the plasmids were present in the *P. sojae* transformants. The genomic DNA was extracted from the transformants and PCR was performed using the primers F and R listed in Supplementary Table 4. **c** Relative transcript levels of *eGFP-PsTET3* overexpressing *P. sojae* transformants. Total RNA was extracted from transformants. Relative transcript levels were determined by RT-qPCR. *PsActin* was used as the internal reference gene. Levels of *PsTET3* were normalized to *PsActin* then set relative to the levels of the wild type (set to 1). Mean values (±SD) of three replicates are shown. Statistical analyses were performed using Two-tailed Student's *t* test. **d** NTA analysis of EV levels released into culture media by eGFP-PsTET3 overexpressing transformants and wild type. Mean values (±SD) of three replicates are shown. Statistical analyses were performed using Two-tailed Student's *t* test. **e** Protein concentration of EVs by eGFP-PsTET3 overexpressing transformants and wild type. Mean values (±SD) of three replicates are shown. Statistical analyses were performed using Two-tailed Student's *t* test. **f, g** eGFP-PsTET3

overexpressing *P. sojae* transformants exhibit reduced virulence. Etiolated soybean hypocotyls were inoculated with zoospore suspensions from the wild-type (P6497), eGFP-PsTET3 overexpressing transformants and eGFP overexpressing transformants as control. **f** Disease symptoms photographed at 2 days post-inoculation. **g** Relative pathogen biomass in inoculated etiolated hypocotyls measured as the ratio between the amounts of *P. sojae* DNA and soybean DNA assayed at 2 dpi by qPCR; levels in P6497-inoculated soybean were set to 1.0. Mean values (±SD) of three replicates are shown. Statistical analyses were performed using Two-tailed Student's *t* test. All experiments were repeated three times with similar results. Source data are provided as a Source Data file.

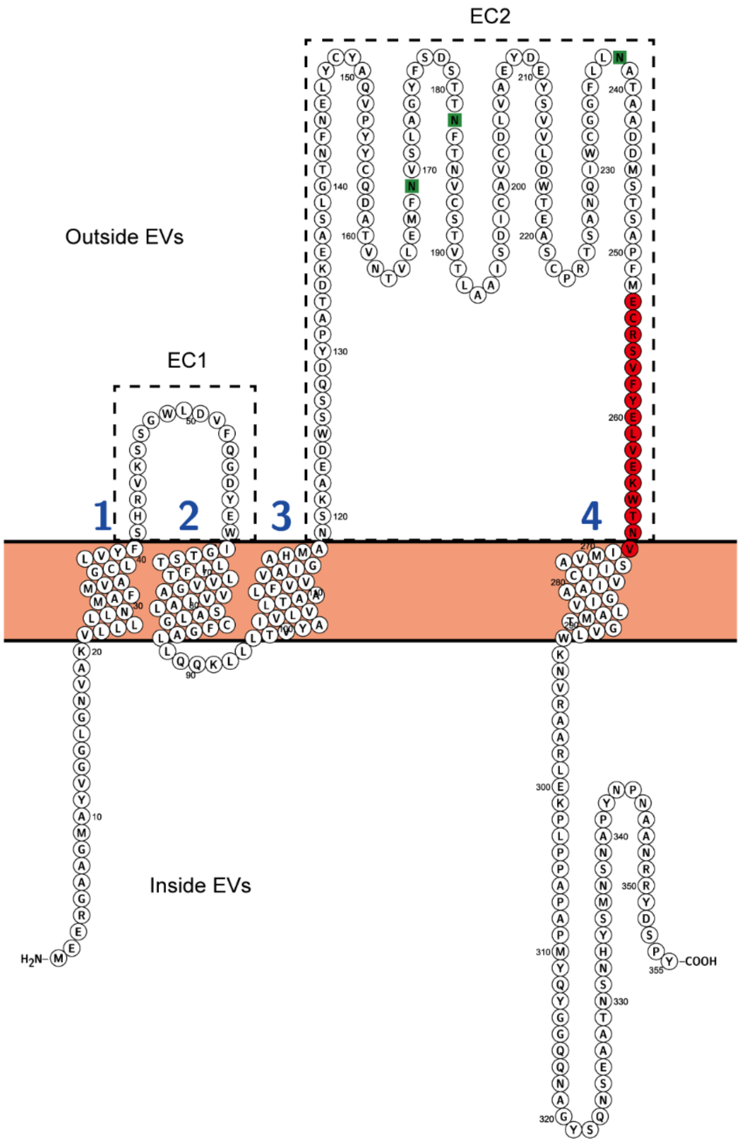

Supplementary Fig. 10 The structure and topology of PsTET3. The images were made using Protter (https://wlab.ethz.ch/protter/start/). EC1 (small extracellular loop) and EC2 (large extracellular loop) were circled by dotted box. The key 16 amino acids in EC2 which are important for induce plant immunity are marked in red.

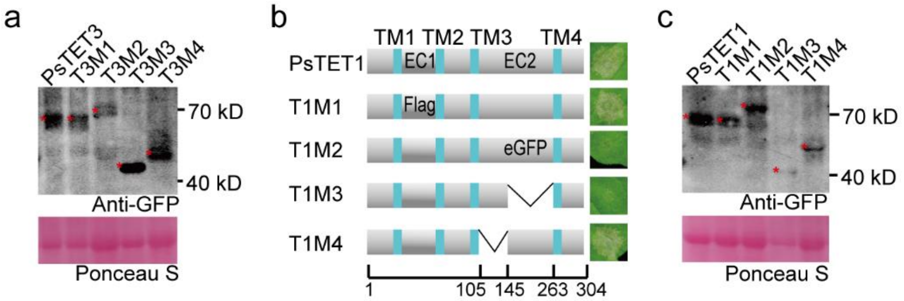

Supplementary Fig. 11 EC2 is required for PsTET1 to induce immune responses. a Immunoblot analysis of PsTET3 and indicated mutants fused with eGFP at the N terminus and transiently expressed in *N. benthamiana* leaves for 2 days. Ponceau S-stained Rubisco protein is shown as a total protein loading control. Red stars indicate expected sizes. **b** Schematic diagram showing the protein structures of PsTET1 and derived deletion or replacement mutants. In T1M1, EC1 was replaced by flag tag. In T1M2, EC2 was replaced by an eGFP tag. In T1M3, 119 residues were deleted from the C-terminus of EC2 (145 to 263). In T1M4, 41 residues were deleted from the N terminus of EC2 (105 to 145). PsTET1 and indicated mutants fused with eGFP at the N terminus. Representative *N. benthamiana* leaves infiltrated with indicated constructs were photographed 3 days after infiltration. **c** Immunoblot analysis of PsTET1 and indicated mutants fused with eGFP at the N terminus and transiently expressed in *N. benthamiana* leaves for 2 days. Ponceau S-stained Rubisco protein is shown as a total protein loading control. Red stars indicate expected sizes. All experiments were repeated three times with similar results. Source data are provided as a Source Data file.

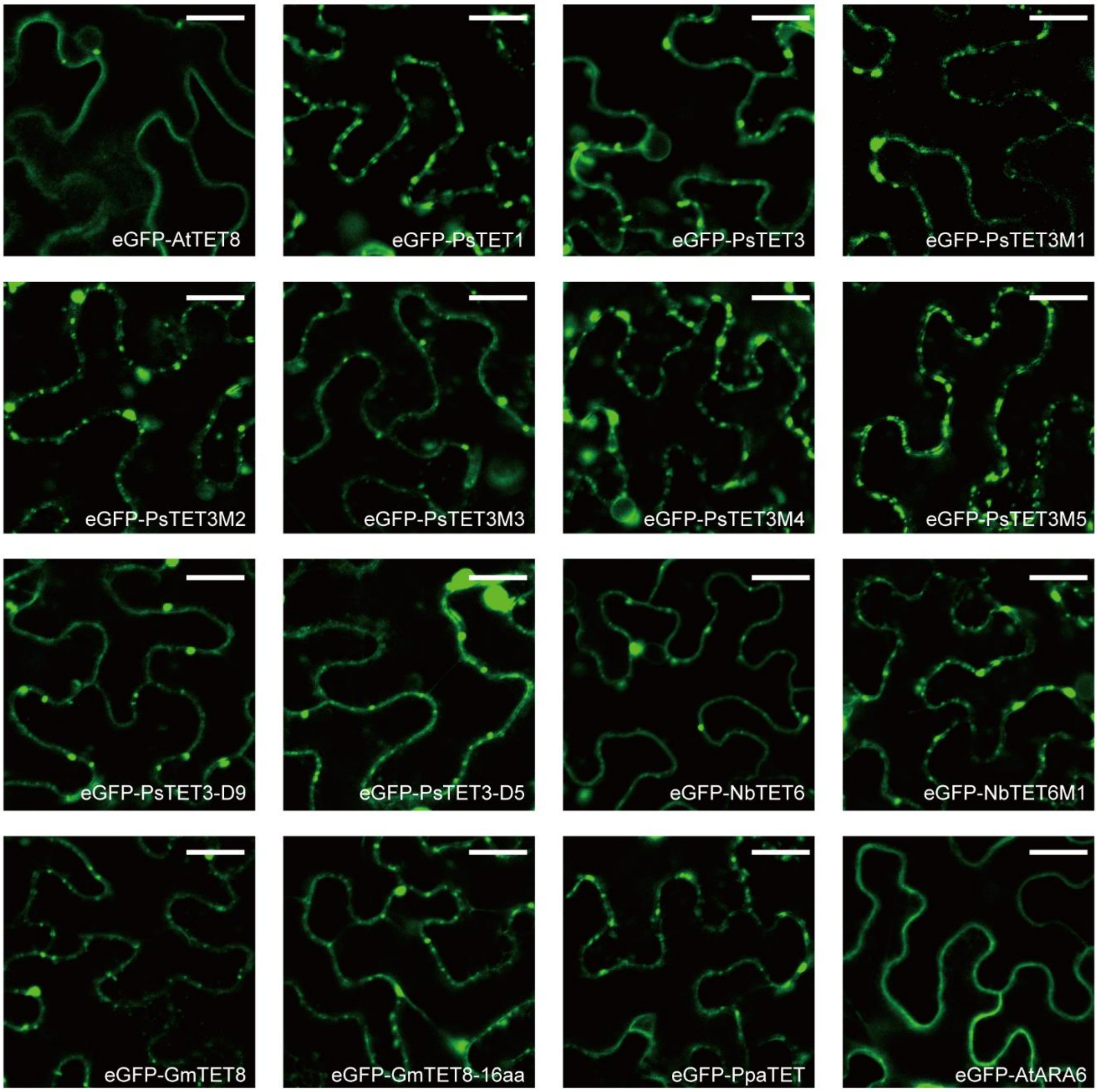

Supplementary Fig. 12 The localization of TET proteins and mutants in *N. benthamiana*. Localization of TET proteins following transient expression in *N. benthamiana*. All TET proteins were fused with an eGFP tag at the N-terminus. *N. benthamiana* leaves infiltrated with indicated constructs were photographed 24 hours after agro- infiltration, prior to the development of cell death. Scale bars, 20 μm. This experiment was repeated three times with similar results.

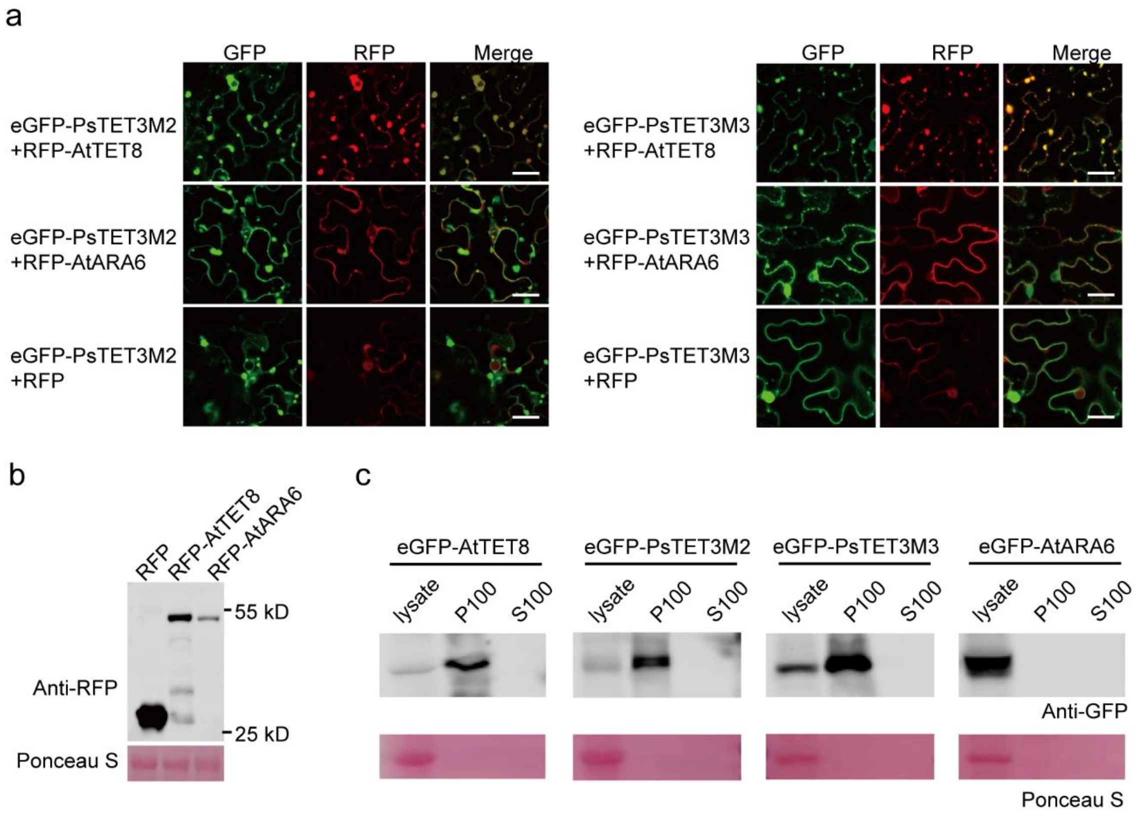

Supplementary Fig. 13 PsTET3 mutants PsTET3M2 and PsTET3M3 localized on *N. benthamiana* EVs. a Colocalization between PsTET3 mutants with MVB marker AtARA6 and EV marker AtTET8. Confocal microscopy images of eGFP-PsTET3M2/eGFP-PsTET3M3 and RFP-AtTET8 and RFP-AtARA6 and RFP as control in *N. benthamiana* leaves. eGFP-PsTET3M2 and eGFP-PsTET3M3 were colocalized with AtTET8 and were partially colocalized with AtARA6. Scale bars, 20 µm**. b** Immunoblot analysis of indicated proteins transiently expressed in *N. benthamiana* leaves. **c** PsTET3M2 and PsTET3M3 are localized to EVs when expressed in *N. benthamiana*. AtTET8 as a positive control and AtARA6 is a negative control. Ponceau S-stained Rubisco protein is shown as a total protein loading control. All experiments were repeated three times with similar results. Source data are provided as a Source Data file.

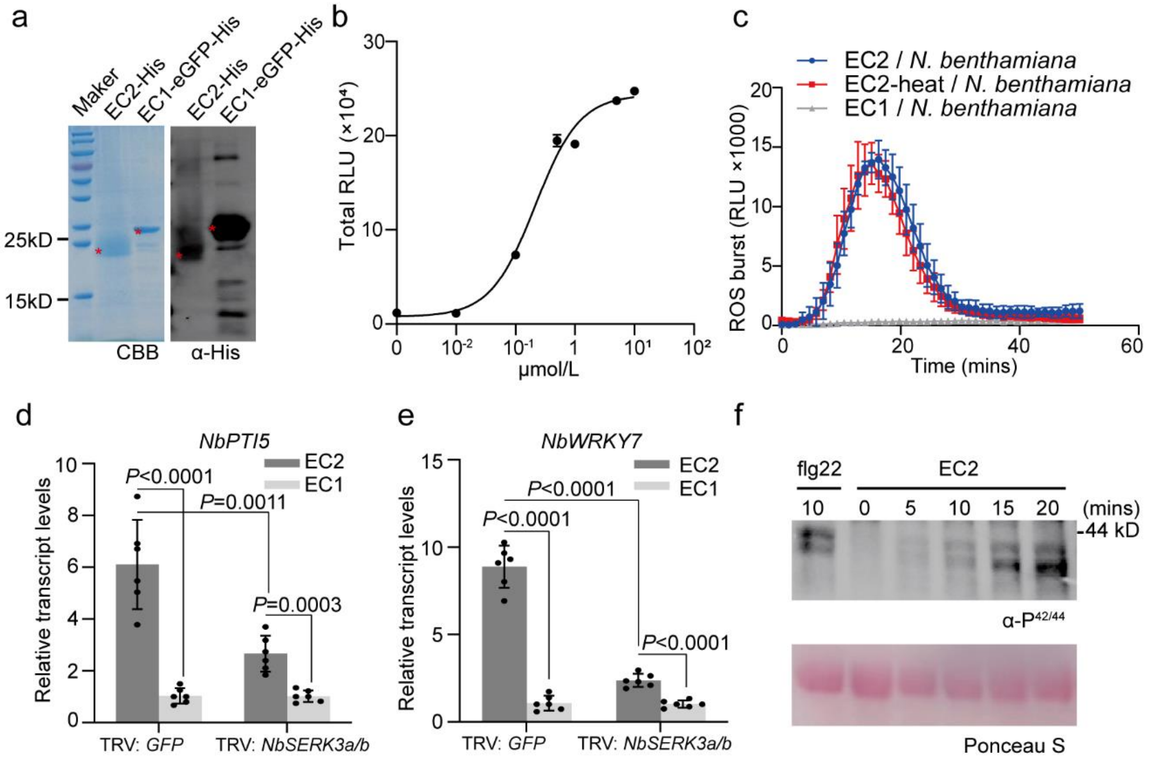

Supplementary Fig. 14 EC2 is the key region for PsTET3 to induce immune responses. **a** C-terminal-His-tagged EC2 protein (PsTET3 residues 116 to 268) and C-terminal-eGFP-His-tagged EC1 (PsTET3 residues 41 to 65) expressed in *P. pastoris*. Western blot and CBB staining of the gel to visualize EC1 and EC2 protein are shown. **b** Dose-response relationship for EC2-induced ROS in *N. benthamiana* leaves. A concentration gradient of EC2 protein (0.01μM, 0.1μM, 0.5μM, 1μM, 5μM, 10μM) was tested. The total RLU was calculated. Mean values (±SD) of three replicates are shown. **c** Production of reactive oxygen species (ROS) in *N. benthamiana* leaf discs treated with 1 μM His-tagged EC2 protein produced in *P. pastoris* or heat-treated EC2 protein or control (EC1-eGFP-His). Mean values (±SD) of three replicates are shown. **d, e** Relative transcript levels of PTI-marker genes in TRV: *GFP* or TRV: *NbSERK3a/b N. benthamiana* leaves after infiltration with 1 μM His-EC2 protein or control (EC1-eGFP-His). Total RNA was extracted 3 hours after treatment. Relative transcript levels of *NbPTI5* and *NbWRKY7* were determined by quantitative reverse transcription PCR. *NbEF1α* was used as the internal reference gene. Relative expression of each marker gene was normalized to *NbEF1α* then set relative to the levels of the buffer control (set to 1.0). Mean values (±SD) of six measurements are

shown. Statistical analyses were performed using Two-tailed Student's *t* test. **f** MAPK phosphorylation triggered by EC2-His protein in soybean leaves. Leaf discs were incubated with 1 μM EC2-His protein or control (EC1-eGFP-His) for 5-20 min. Total protein was extracted and analyzed by immunoblotting using antibodies against phospho-p42/44 MAPK. Ponceau S-stained Rubisco protein is shown as a total protein loading control. All experiments were repeated three times with similar results. Source data are provided as a Source Data file.

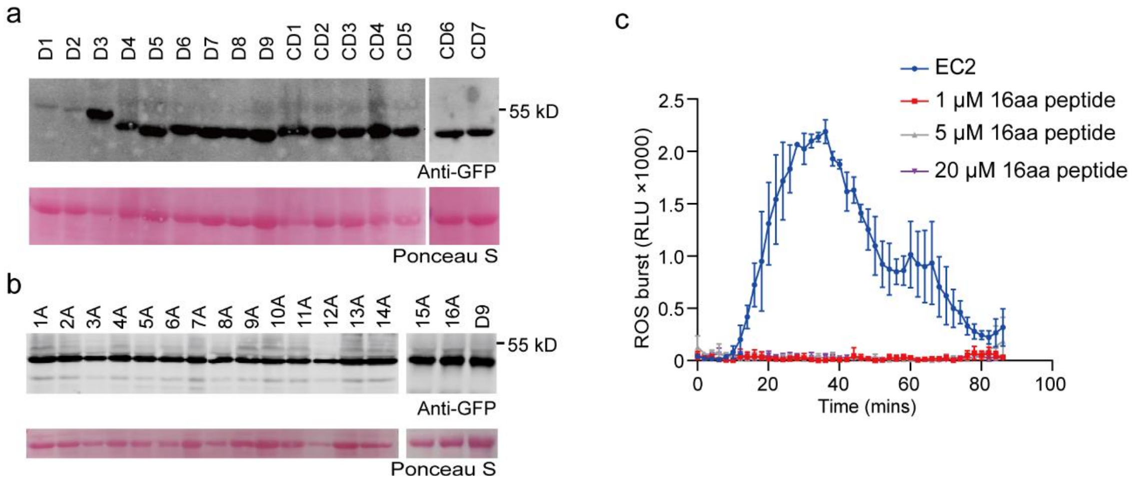

Supplementary Fig. 15 Levels of PsTET3 mutant proteins after transient expression in *N. benthamiana* leaves. a, b Immunoblot analysis of eGFP-tagged mutant proteins in total proteins extracted from *N. benthamiana* leaves 2 days after agro-infiltration. Ponceau S-stained Rubisco protein is shown as a total protein loading control. **c** Production of reactive oxygen species (ROS) in *N. benthamiana* leaf discs treated with 1 μM His-tagged EC2 protein produced in *P. pastoris* or different concentration (1μM, 5μM, 20μM) of synthetic 16aa peptide. Mean values (±SD) of three replicates are shown. These experiments were repeated three times with similar results. Source data are provided as a Source Data file.

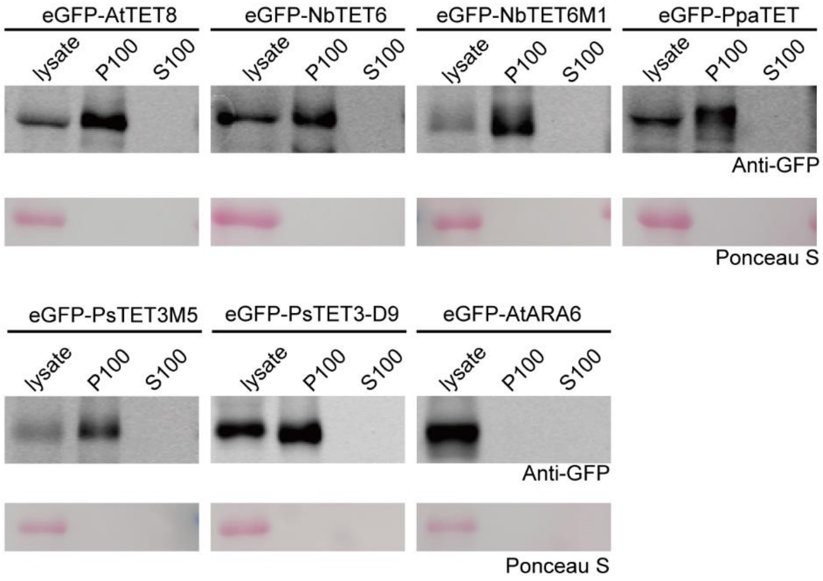

Supplementary Fig. 16 Heterologous TET proteins can target *N. benthamiana* EVs when transiently expressed in *N. benthamiana*. The *N. benthamiana* EVs were isolated 2 days after infiltration, prior to the development of cell death. EV proteins were detected by western blotting with anti-GFP antibodies. AtTET8 was used as a positive control and AtARA6 as a negative control. Ponceau S-stained Rubisco protein is shown as a total protein loading control. This experiment was repeated three times with similar results. Source data are provided as a Source Data file.

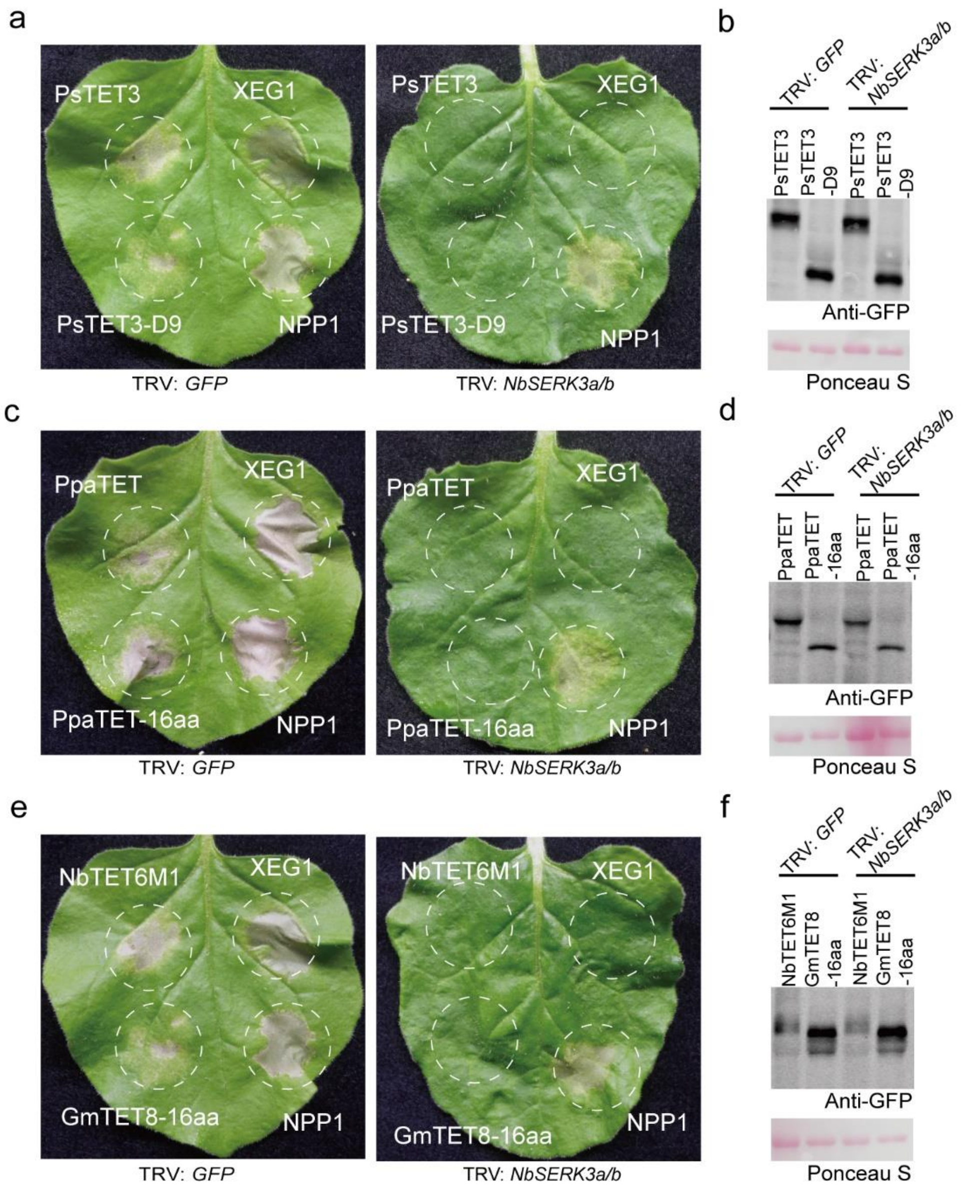

Supplementary Fig. 17 The cell death induced by TET mutant proteins also is NbSERK3a/b-dependent. a, c, e Representative leaves showing cell death induced by expression of the indicated proteins in TRV: *GFP* or TRV: *NbSERK3a/b N. benthamiana* leaves. Leaves (n=9) were photographed three days after agro-infiltration. **b, d, f** Immunoblot analysis of transiently expressed TET proteins fused with an eGFP tag at the N-terminus in total protein extracts from TRV: *GFP* or TRV: *NbSERK3a/b N. benthamiana* leaves 2 days after agro-infiltration. Ponceau S-stained Rubisco protein is shown as a total protein loading control. Correct targeting of PsTET3-D9, PpaTET, and

GmTET8-16aa is documented in Supplementary Fig. 12. All experiments were repeated three times with similar results. Source data are provided as a Source Data file.

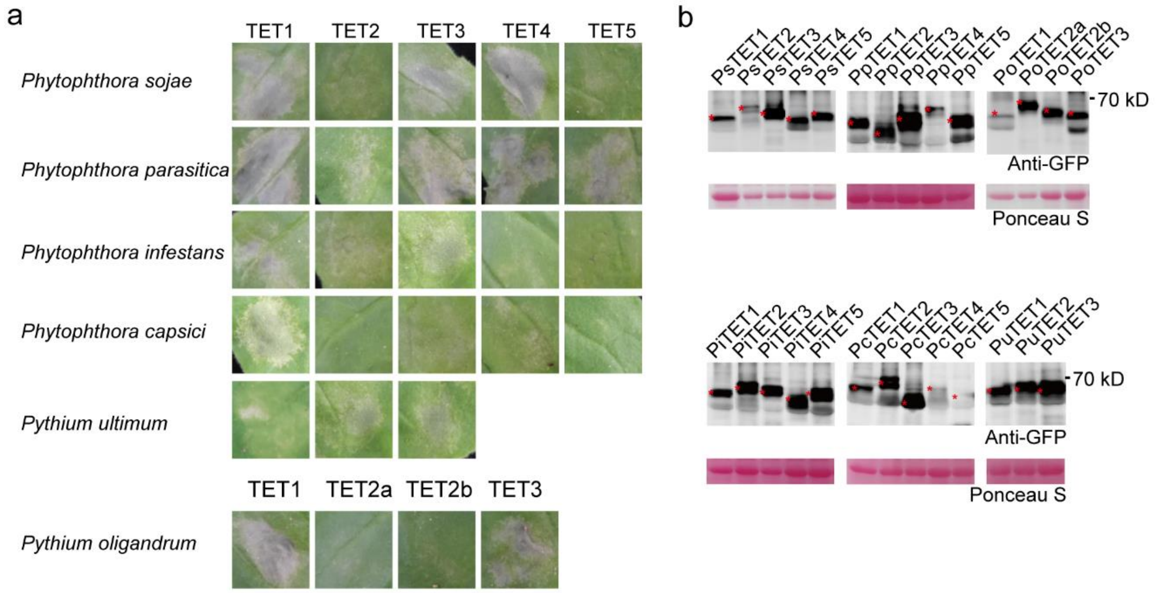

Supplementary Fig. 18 Cell death induced by TET proteins from different oomycetes expressed in *N. benthamiana* leaves. a Representative leaves showing whether eGFP-fused TET proteins from the indicated species of oomycetes could induce cell death in *N. benthamiana* leaves. Leaves (n=9) were photographed three days after agro-infiltration. **b** Western blot analysis using anti-GFP antibody to detect the accumulation of each TET protein in total leaf proteins 2 days after agro-infiltration of *N. benthamiana* leaves. Ponceau S-stained Rubisco protein is shown as a total protein loading control. All experiments were repeated three times with similar results. Source data are provided as a Source Data file.

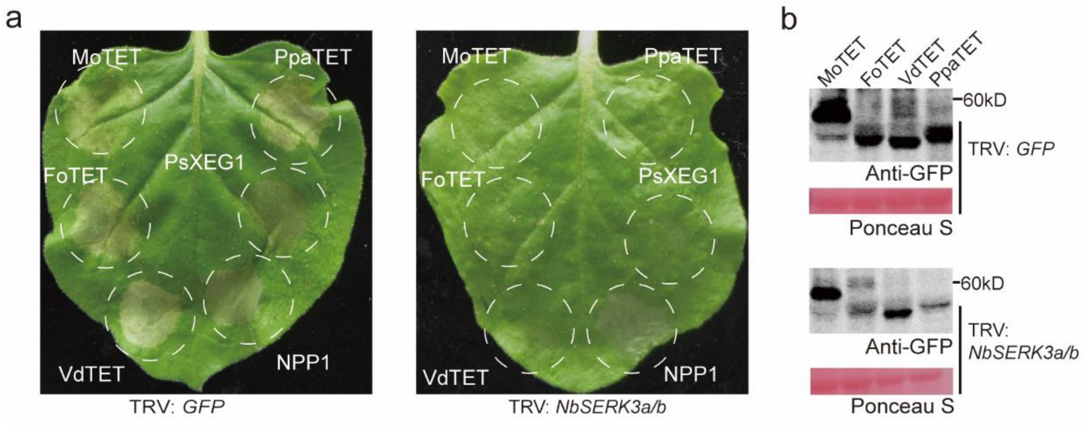

Supplementary Fig. 19 The cell death induced by TET proteins from fungal pathogens is dependent on NbSERK3a/b in *N. benthamiana*. a Representative leaves showing cell death induced by expression of the indicated proteins in TRV: *GFP* or TRV: *NbSERK3a/b N. benthamiana* leaves. Leaves (n=9) were photographed three days after agro-infiltration. **b** Immunoblotting analysis of transiently expressed TET proteins fused with an eGFP tag at the N terminus in total protein extracts from TRV: *GFP* or TRV: *NbSERK3a/b N. benthamiana* leaves 2 days after agro-infiltration. Ponceau S-stained Rubisco protein is shown as a total protein loading control. Mo = *Magnaporthe oryzae*; Fo = *Fusarium oxysporum*; Vd = *Verticillium dahliae*; Ppa = *Phakopsora pachyrhizi*. All experiments were repeated three times with similar results. Source data are provided as a Source Data file.

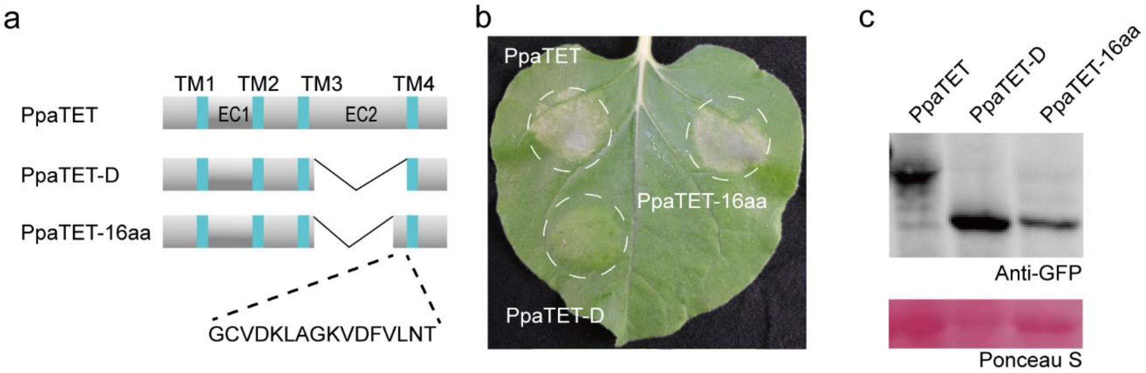

Supplementary Fig. 20 The C-terminus of EC2 is required for the Pls1 protein from the fungus *P. pachyrhizi* (PpaTET) to induce cell death in *N. benthamiana*. a Schematic diagram showing the protein PpaTET, its EC2 deletion mutant and the deletion retaining the C-terminal 16 residues of EC2. **b** Cell death triggered by indicated constructs in *N. benthamiana*. Representative *N. benthamiana* leaves were photographed 3 days after infiltration. **c** Immunoblot analysis of transiently expressed constructs in *N. benthamiana* leaves. Total leaf proteins were extracted 2 days after agro-infiltration. Ponceau S-stained Rubisco protein is shown as a total protein loading control. Correct targeting of PpaTET is documented in Supplementary Figs. 12 and 16. All experiments were repeated three times with similar results. Source data are provided as a Source Data file.

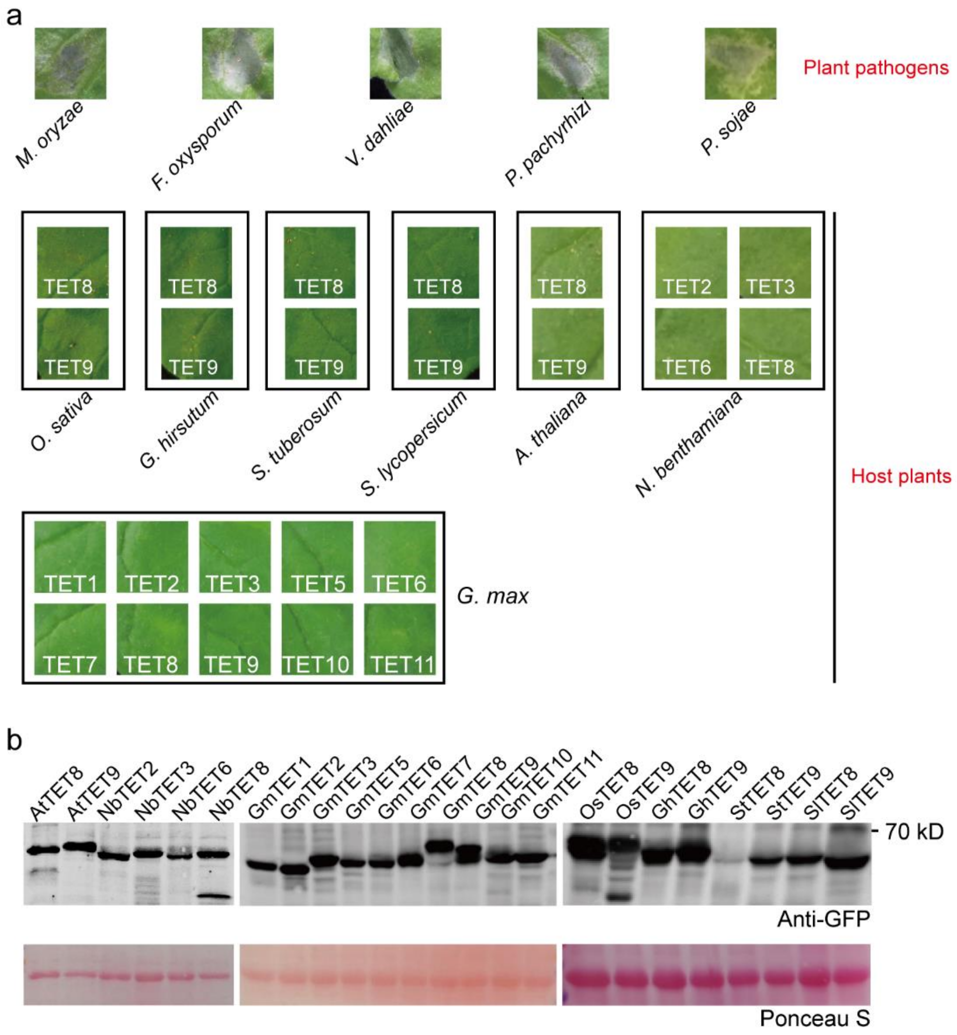

Supplementary Fig. 21 TET proteins derived from plants could not induce cell death in *N. benthamiana*. a Representative leaves showing TET proteins from plants could not induce cell death in *N. benthamiana,* in contrast to fungal and oomycete TET proteins. Leaves (n=9) were photographed three days after agro-infiltration. **b** Immunoblot analysis of transiently expressed TET proteins fused with an eGFP tag at the N-terminus in *N. benthamiana* leaves. Total leaf proteins were extracted 2 days after agro-infiltration. Ponceau S-stained Rubisco protein is shown as a total protein loading control. All experiments were repeated three times with similar results. Source data are provided as a Source Data file.

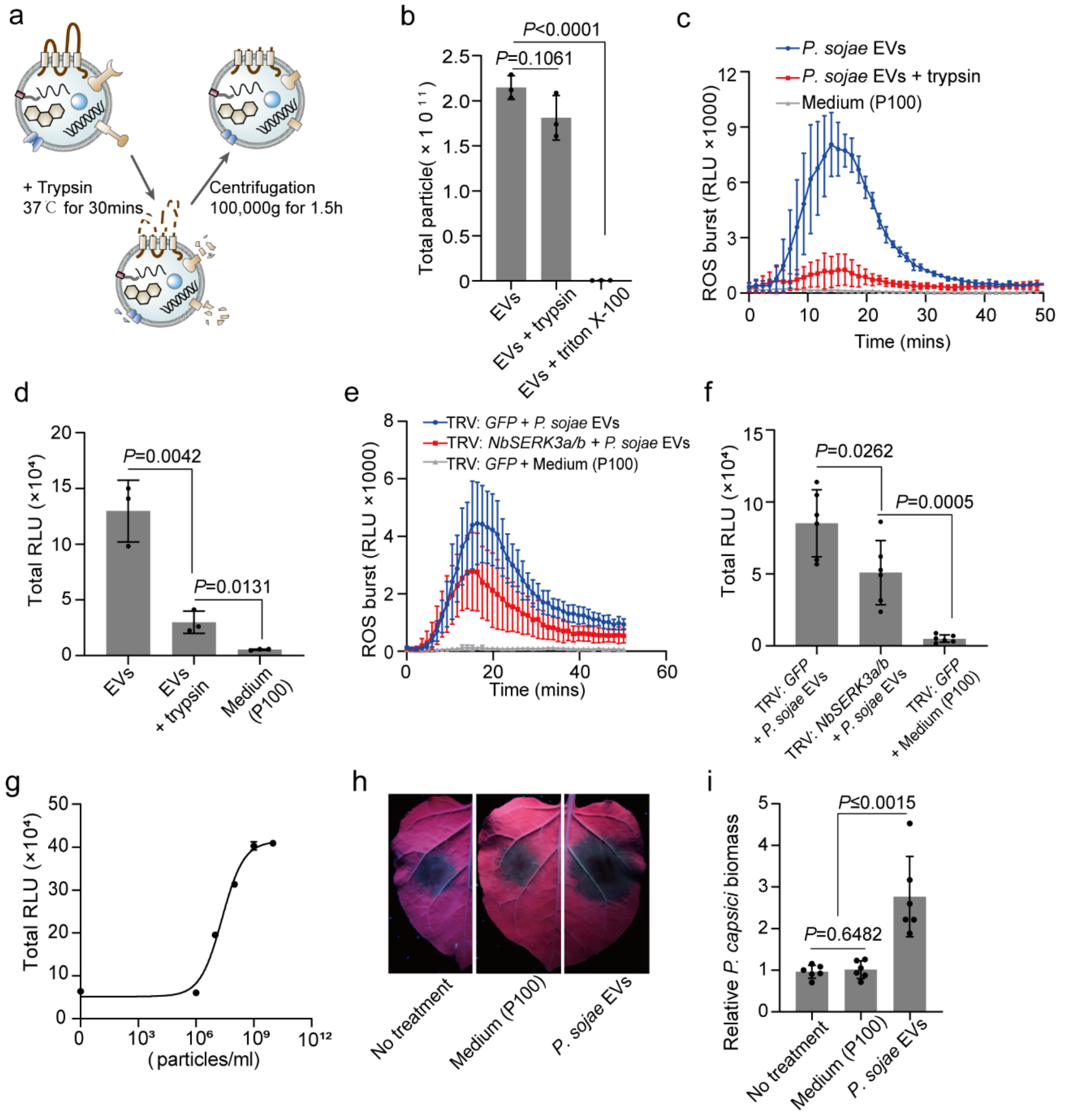

Supplementary Fig. 22 The function analysis of EVs released by *P. sojae*. a Schematic diagram showing isolation of EVs by ultracentrifugation after trypsin digestion. **b** NTA analysis of EV levels after treated with trypsin or triton X-100. Mean values (±SD) of three replicates are shown. Statistical analyses were performed using Two-tailed Student's *t* test. **c, d** Production of reactive oxygen species (ROS) in *N. benthamiana* leaf discs treated with purified EVs of *P. sojae* or EVs pretreated with trypsin or medium P100. Mean values (±SD) of three replicates are shown. Statistical analyses were performed using Two-tailed Student's *t* test. **e, f** Production of ROS in TRV: *GFP* or TRV: *NbSERK3a/b N. benthamiana*

leaf discs treated with *P. sojae* EVs or control (medium P100). Mean values (±SD) of three replicates are shown. Statistical analyses were performed using Two-tailed Student's *t* test. **g** Dose-response relationship for *P. sojae* EVs-induced ROS in *N. benthamiana* leaves. A concentration gradient of *P. sojae* EVs was tested. The total RLU was calculated. Mean values (±SD) of three replicates are shown. **h** Pretreated with low concentration of *P. sojae* EVs promote the infection of *P. capsici* in *N. benthamiana*. 10^6 particle ml^{-1} *P. sojae* EVs and control (medium P100) were infiltrated in *N. benthamiana* leaves, followed by inoculation with *P. capsici*. Infected leaves were photographed at 48h after inoculation. **i** Relative pathogen biomass in inoculated *N. benthamiana* measured as the ratio between the amounts of *P. capsici* DNA and *N. benthamiana* DNA assayed at 2 dpi by qPCR; levels in control treated were set to 1.0. Mean values (±SD) of six measurements are shown. Statistical analyses were performed using Two-tailed Student's *t* test. All experiments were repeated three times with similar results. Source data are provided as a Source Data file.

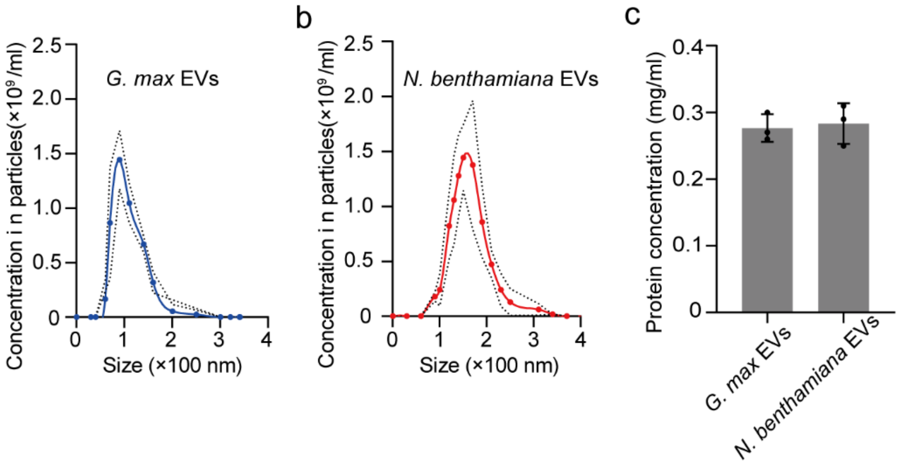

Supplementary Fig. 23 Nanoparticle tracking analysis of EVs released by *N. benthamiana* and *G. max*. a, b Nanoparticle tracking analysis of purified EVs from leaf apoplasts of **(a)** *G. max* and **(b)** *N. benthamiana*. Mean values (±SD) of three replicates are shown. **c** Protein concentration of EVs by *G. max* and *N. benthamiana*. **c** Protein concentration of EVs by *G. max* and *N. benthamiana*. Mean values (±SD) of three replicates are shown. All experiments were repeated three times with similar results. Source data are provided as a Source Data file.
