# Supplementary material for: Divergent sequences of tetraspanins enable plants to specifically recognize microbe-derived extracellular vesicles
Source: Nat Commun. 2023 Aug 12;14:4877. doi: 10.1038/s41467-023-40623-0 (PMC10423219; doi:10.1038/s41467-023-40623-0)
Supplement: Supplementary file 3 — Description of Additional Supplementary Files [file 41467_2023_40623_MOESM3_ESM.pdf]

Description of Additional Supplementary Files

Supplementary Data 1. LC-MS data for *P. sojae* EVs-associated proteins. Related to Figure 1.

Supplementary Data 2. Transcription levels of 95 genes. Related to Figure 1.

Supplementary Data 3. The amino acid sequences of PsTET3 and truncated mutants.

Supplementary Data 4. Sequence information of PsTETs.

Supplementary Data 5. Primers used in this study.
