# Supplementary material for: Divergent sequences of tetraspanins enable plants to specifically recognize microbe-derived extracellular vesicles
Source: Nat Commun. 2023 Aug 12;14:4877. doi: 10.1038/s41467-023-40623-0 (PMC10423219; doi:10.1038/s41467-023-40623-0)
Supplement: Supplementary file 9 — Reporting Summary [file 41467_2023_40623_MOESM9_ESM.pdf]

nature portfolio

Corresponding author(s): Yuanchao Wang

Last updated by author(s): Jul 20, 2023

Reporting Summary

Nature Portfolio wishes to improve the reproducibility of the work that we publish. This form provides structure for consistency and transparency in reporting. For further information on Nature Portfolio policies, see our Editorial Policies and the Editorial Policy Checklist.

Statistics

For all statistical analyses, confirm that the following items are present in the figure legend, table legend, main text, or Methods section.

n/a	Confirmed	
☐	☒	The exact sample size (*n*) for each experimental group/condition, given as a discrete number and unit of measurement
☐	☒	A statement on whether measurements were taken from distinct samples or whether the same sample was measured repeatedly
☐	☒	The statistical test(s) used AND whether they are one- or two-sided<br>*Only common tests should be described solely by name; describe more complex techniques in the Methods section.*
☒	☐	A description of all covariates tested
☒	☐	A description of any assumptions or corrections, such as tests of normality and adjustment for multiple comparisons
☐	☒	A full description of the statistical parameters including central tendency (e.g. means) or other basic estimates (e.g. regression coefficient) AND variation (e.g. standard deviation) or associated estimates of uncertainty (e.g. confidence intervals)
☐	☒	For null hypothesis testing, the test statistic (e.g. *F*, *t*, *r*) with confidence intervals, effect sizes, degrees of freedom and *P* value noted<br>*Give P values as exact values whenever suitable.*
☒	☐	For Bayesian analysis, information on the choice of priors and Markov chain Monte Carlo settings
☒	☐	For hierarchical and complex designs, identification of the appropriate level for tests and full reporting of outcomes
☒	☐	Estimates of effect sizes (e.g. Cohen's *d*, Pearson's *r*), indicating how they were calculated

Our web collection on statistics for biologists contains articles on many of the points above.

Software and code

Policy information about availability of computer code

Data collection	Genomic databases downloaded from NCBI (https://www.ncbi.nlm.nih.gov/)<br>Tetraspanin HMM (PFAM ID: PF00335; downloaded from http://pfam.xfam.org)
Data analysis	Software HMMER (version 3.0; with default parameter); SMART (http://smart.embl-heidelberg.de); TMHMM Server v. 2.0 (http://www.cbs.dtu.dk/services/TMHMM-2.0/); ClustalW2 program; MEGA7; GraphPad Prism 8.4.3; PhyML v3.0.

For manuscripts utilizing custom algorithms or software that are central to the research but not yet described in published literature, software must be made available to editors and reviewers. We strongly encourage code deposition in a community repository (e.g. GitHub). See the Nature Portfolio guidelines for submitting code & software for further information.

Data

Policy information about availability of data

All manuscripts must include a data availability statement. This statement should provide the following information, where applicable:

- Accession codes, unique identifiers, or web links for publicly available datasets
- A description of any restrictions on data availability
- For clinical datasets or third party data, please ensure that the statement adheres to our policy

The data that support the findings of this study are available in the Supplementary Information and Source data are provided with this paper. The mass spectrometry proteomics raw data have been deposited to the ProteomeXchange Consortium via the PRIDE partner repository with the dataset identifier

PXD040458 [https://www.ebi.ac.uk/pride/archive/projects/PXD040458] .

Human research participants

Policy information about studies involving human research participants and Sex and Gender in Research.

Reporting on sex and gender	N/A
Population characteristics	N/A
Recruitment	N/A
Ethics oversight	N/A

Note that full information on the approval of the study protocol must also be provided in the manuscript.

Field-specific reporting

Please select the one below that is the best fit for your research. If you are not sure, read the appropriate sections before making your selection.

[x] Life sciences [ ] Behavioural & social sciences [ ] Ecological, evolutionary & environmental sciences

For a reference copy of the document with all sections, see nature.com/documents/nr-reporting-summary-flat.pdf

Life sciences study design

All studies must disclose on these points even when the disclosure is negative.

Sample size	Samples sizes were determined based on the generation of convincing and compelling results. At least three biological replicates were performed in all experiments. The number of replicates is indicated in the figure legend and/or in the method section.
Data exclusions	No data were excluded unless the experiment failed.
Replication	Experiments were repeated at least three times.
Randomization	Samples were allocated randomly into experimental groups.
Blinding	Investigators were blinded to the cell death- and pathogenicity-related assays by labeling each strain or treatment with simple, non-informative labels and randomized allocation of mock and infected plants in climate chamber. Investigators were not blinded to western blots because the investigators needed to determine appropriate samples to run in particular lanes of the gel.

Reporting for specific materials, systems and methods

We require information from authors about some types of materials, experimental systems and methods used in many studies. Here, indicate whether each material, system or method listed is relevant to your study. If you are not sure if a list item applies to your research, read the appropriate section before selecting a response.

Materials & experimental systems

n/a	Involved in the study	
[ ]	[x]	Antibodies
[x]	[ ]	Eukaryotic cell lines
[x]	[ ]	Palaeontology and archaeology
[x]	[ ]	Animals and other organisms
[x]	[ ]	Clinical data
[x]	[ ]	Dual use research of concern

Methods

n/a	Involved in the study	
[x]	[ ]	ChIP-seq
[x]	[ ]	Flow cytometry
[x]	[ ]	MRI-based neuroimaging

Antibodies

Antibodies used	Anti-GFP(Mouse antibody; 1:5000; Abmart #M20004L); Anti-RFP(Mouse antibody; 1:2000; ChromoTek #6G6); Anti-His(Mouse antibody; 1:5000; Abmart #M20001L); IRDye 800CW Goat anti-Mouse IgG(H+L) antibody(926-32210, LI-COR, 1:10000); Anti-phospho-p44/42 MAPK(Erk1/2) antibody(Cell Signaling, 1:5000, #4370s) and IRDye 800CW Goat anti-Rabbit IgG(H+L) antibody (926-32211, LI-COR, 1:10000)

Validation	All antibodies recognized tagged proteins, though unspecific bands were also detected. Anti-GFP and Anti-RFP were used to validate the expression of tagged proteins in N. benthamiana and P. soaje. Anti-His was used to detect the purified proteins by Pichia pastoris. Anti-phospho-p44/42 MAPK (Erk1/2) antibody was used to validate the phosphorylation of MAPK proteins in soybean and N. benthamiana.
